# Quantitative NMR (qNMR) spectroscopy based investigation of the absolute content, stability and isomerization of 25-hydroxyvitamin D2/D3 and 24(*R*),25-dihydroxyvitamin D2 in solution phase

**DOI:** 10.1038/s41598-022-06948-4

**Published:** 2022-02-22

**Authors:** Neeraj Singh, Judith Taibon, Stephan Pongratz, Christian Geletneky

**Affiliations:** Roche Diagnostics, Nonnenwald 2, 82377 Penzberg, Germany

**Keywords:** Analytical chemistry, Organic chemistry

## Abstract

Vitamin D is an important parameter, in serum/plasma based diagnostic analysis, for the determination of optimal regulation of calcium and phosphate homeostases in the human body, vital for the monitoring/progression of osteomalacia and rickets. Particularly, the quantification of 25-hydroxyvitamin D2, 25-hydroxyvitamin D3 and 24*R*,25-dihydroxyvitamin D in blood is an excellent indicator for the vitamin D status of a patient. For this purpose, LC–MS/MS methods, based on appropriate vitamin D reference standards, are considered to be ‘gold standard’ for such measurements. We have utilized quantitative NMR spectroscopy to determine the absolute content of these molecules, available as non-certified chemicals, and have determined the stability of these callibrators in borderline polar solvents at room temperature. We have observed significant isomerization of the analytes, which can play a big role in quantification of these analytes by hyphenated LC and GC analytical techniques. Appropriate explanations are given for the observation of new impurities with time in solution phase. The spin system selected for quantitation was determined using relevant 1D and 2D NMR pulse sequences. The advantage of the qNMR approach is that it is based on the quantification of atoms rather than molecular properties (e.g., quantitation by LC/UV, GC, etc.). Since the signals in an NMR spectrum are different nuclear spin-systems dispersed precisely in a magnetic environment, with the intensity being directly proportional to the amount of a particular type of nuclear spin, this technique delivers unparalleled information about the chemical structure and the absolute content.

Vitamin D^1^ constitutes a group of lipophilic secosteroids, which play a pivotal role in the upkeep of musculoskeletal system by regulating calcium and phosphate absorption in the intestine. Recently, the role of circulating vitamin D levels has been implicated in infectious diseases, hemodynamic ailments and specific cancers^1^. Although, dietary pills available for general public includes only vitamin D2 (ergocalciferol) and vitamin D3 (cholecalciferol), these are in fact pro-hormones which are hydroxylated at the side chain, in vivo, to give the corresponding 25-hydroxy analogues, which are the surrogate molecules responsible for physiological response, owing to their longer half-life compared to the biologically active 1*α*,25-dihydroxyvitamin D^1^. The concentration of these analytes, viz., 25-hydroxyvitamin D2 (1, ercalcidiol) and 25-hydroxyvitamin D3 (4, calcidiol) (Fig. 1), is the basis of diagnosis for the above mentioned disorders^2^. Moreover, the quantification of 24,25-dihydroxyvitamin D, which is a product of the catabolism of 25-hydroxyvitamin D, is an excellent indicator of the catabolic efficiency of CYP24A1^2^. Nowadays, quantification of analytes by ID-LC–MS/MS based methods^3^ are considered to be the ′gold standard′, however, in biological matrices. Vitamin D solution based reference standards (primary callibrators)^4^ are available from some metrological institutes, although in some cases, the entire concentration range is not covered for a particular physiological disorder. This leads to a demand for highly characterized solid vitamin D standards, which can be utilized as per convenience. However, currently, there are no such solid standards available.

**Figure 1:**
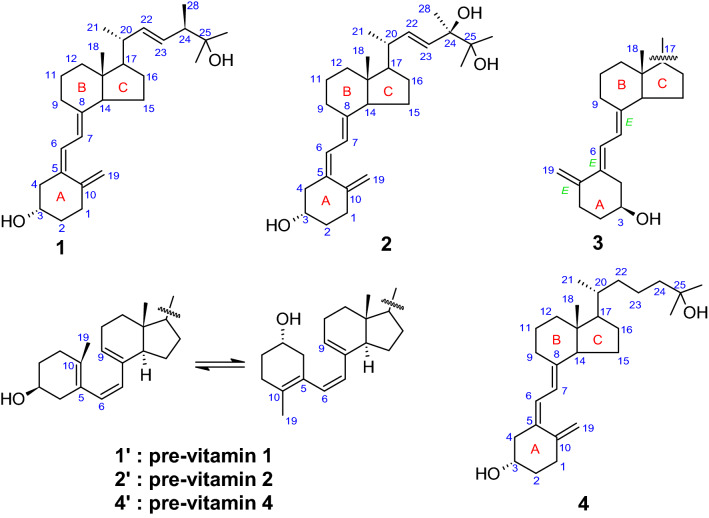
Structures of Vitamin D vitamers ((25-hydroxyvitamin D2 (1); 24*R*,25-dihydroxyvitamin D2 (2); 25-dihydroxyvitamin D3 (4)) and the corresponding isomerization pre-vitamin products.

Structurally, vitamin D vitamers are characterized by the presence of a typical hexatriene system, the configuration of which depends upon the analogue in question^5–13^. Depending upon this configuration, a lot of reactions are possible in solution, viz., cycloaddition, electrocyclic cyclization, sigmatropic shifts, H-abstraction, etc., in solution phase^5^. Additionally, the effect of free-radicals, singlet oxygen, pH of the solution, temperature and sunlight can also lead to the formation of unwanted species^5–14^, which can lead to highly erroneous results, when such solutions are utilized as primary calibrators.

Owing to the afore-mentioned information, we decided to study the stability of 25-hydroxyvitamin D2 (1) and D3 (4), along with 24(*R*),25-dihydroxyvitamin D2 (2) by quantitative NMR spectroscopy. It must be noted that the quantification of 25-hydroxyvitamin D3 (4) and 25-hydroxyvitamin D2 (1), by qNMR methodology, has been published elsewhere, however, in methanol^15^. Our aim is to characterize normal synthetic vitamin D compounds (non-reference standards) and to utilize qNMR in real-world conditions.

Moreover, Nuclear Magnetic Resonance (NMR) is an established technique for investigating the structure of small molecules, proteins, nucleic acids, carbohydrates, polymers, etc. A typical NMR spectrum (for example, ^1^H NMR) represents all the chemically and magnetically different hydrogen atoms present in the molecule, and the intensity of an NMR signal is directly proportional to the amount or ′counts′ of the resonant nuclei. Owing to the above properties, quantitative NMR or qNMR is very well suited for a primary ratio quantitative method^16–18^, and with the introduction of NIST PS1 Benzoic Acid qNMR standard^18^, the measurements will be directly traceable to the SI units. Recent efforts by academia, metrological institutes (NIST, NMIJ, NMIA, etc.), pharmacopoeia (USP, EP and BP), and chemical companies (Sigma-Aldrich (Merck), Wako) to quantify small molecules with quantitative NMR technique, with traceability to NIST SRM Benzoic Acid 350b (coulometric)/NIST PS1 qNMR internal standard, have been readily accepted by the scientific/industrial community worldwide owing to the ease and accuracy of the method.

## Materials and methods

25-Hydroxyvitamin D2 (1, Cat No. HY-3249, Batch No. 16275), 25-Hydroxyvitamin D3 (4, Cat No.HY-32351A/CS-0847, Batch No. 43822) were purchased from MedChem Express. 24(*R*),25-dihydroxyvitamin D2 (2, Catalogue No. ENA250, Batch No. MAB1473) was purchased from Endotherm. Chloroform-D (Product No. 416754) was purchased from SigmaAldrich GmbH Germany. 3 mm NMR tubes equipped with Teflon-caps were purchased from SigmaAldrich GmbH Germany. qNMR internal standard, 1,2,4,5-tetrachloronitrobenzene (TraceCert, Product No. 40384), was also purchased from SigmaAldrich GmbH Germany.

Weighing was done on a Mettler-Toledo XPR6U Ultra-Microweighing balance. NMR measurements were performed on a JEOL 500 MHz NMR equipped with a N_2_-cooled Super-Cool cryo-probe-head (ca. 2–3 times the sensitivity of a RT probe-head). ^13^C-decoupled sequence was utilized for the quantification of all the analytes. Spectra are referenced to chloroform signal (7.27 ppm). A spin–lattice relaxation time of 70 s (> 5 T1) was used for all the quantitative ^1^H NMR measurements. Experiments were performed at 300 K. 2D experiments along with JRes spectra were acquired with standard pulse sequences on a JEOL 600 MHz NMR spectrometer equipped with a Royal Probe. qNMR experiments for absolute content determination were completed within 90 min after dissolving the analyte-internal standard mixture in chloroform-D.

## Results and discussion

### Choice of solvent

Chloroform-D was used as a solvent of choice owing to the good dispersion of signals along with it being a border-line polar solvent (on *E*_T_(30), Z and π* solvent scales). We chose CDCl_3_ because it does not induce any solvent mediated effects on the reactions and isomerization of vitamin D, along with ensuring excellent solubility and the border-line properties would enable to assess the true nature of vitamin D2/D3 isomerization. Moreover, the structure of the cybotactic sphere created with vitamin D/CDCl_3_ would not lead to a significant alteration of the charge density, thereby, preserving the true nature of vitamin D reactivity. On the other hand, utilization of polar deuterated solvents, would lead to exchange of labile protons, and thus a kinetic isotope effect might affect the rate of isomerization. Moreover polar solvents have a tendency to stabilize or destabilize transition states owing to their high dielectric constant.

### 25-Hydroxyvitamin D2

In case of 25-hydroxyvitamin D2 (1), the most downfield proton at *δ* = 6.24 ppm (^3^*J* = 11.17 Hz, 1H, H-6) was utilized for quantification (Fig. 1). The structure assignment of this quantifiable proton resonance was ascertained by JRes, 2D-TOCSY and 2D-NOESY experiments (For details, see Supporting Information). Absence of cross-peak for the geminal vinylidene protons clearly confirms the structure assignment for H-6. Additionally, it can be seen from JRes spectrum, that the splitting is not a doublet (d), but a doublet of triplets (dt) owing to long-range coupling with six-membered ring protons. The 2D spectra were measured after ca. 56 h in order to ascertain the structure of **1** as well as the emerging isomers. For absolute content determination, 0.91‒1.01 mg of 1 was weighed together with the qNMR internal standard (1,2,4,5-tetrachloronitrobenzene; 0.83‒1.32 mg) on an ultra-micro weighing balance. To this mixture was added CDCl_3_ (ca. 190 µL, pH neutral), and the solution was transferred to a 3 mm NMR tube. Triplicate measurements were performed to determine the avg. absolute content of **1**, which yields a value of 88% (Table 1).

**Table 1:** Calculation of qNMR absolute content of analytes **1**, **2** and **4**.

Analyte	Quantifiable resonance	Analyte weighed (mg)	ISTD weighed (mg)	qNMR Absolute Content (g/g)
25-Hydroxyvitamin D2	H6	0.9106	1.3173	87.75
25-Hydroxyvitamin D2	H6	1.0101	0.8294	88.17
25-Hydroxyvitamin D2	H6	0.9491	1.3081	88.09
24(*R*),25-Dihydroxyvitamin D2	H6	2.1579	1.0627	93.46
25-Hydroxyvitamin D3	H6	2.4947	1.3631	91.43
25-Hydroxyvitamin D3	H6	1.8509	1.0965	92.35
25-Hydroxyvitamin D3	H6	1.8082	1.0402	91.74

Sample 1 was further utilized for the investigation of stability in solution at RT. No special precautions were taken, to imitate real-world conditions, to protect the sample from light. However, the NMR tube was either in the NMR spectrometer or in the auto-sampler for the entire span of measurements. Measurements were performed after every 8 h to ascertain the short-term stability of Ercalcidiol, to imitate real world conditions in analytical laboratories. A total of 8 measurements were performed, including the one at T_0_, in a time-span of 56 h. A total loss of 5.46% was observed in the absolute content of molecule **1**, which is quite a significant loss, if considered for highly precise LC–MS/MS analytics.

The nature of emerging signals correspond to that of pre-25-hydroxyvitamin D2^19^, the structural conformation of which can be represented as an equilibrium of c*Z*c (s-*cis*, s-*cis*) and t*Z*c (s-*trans*, s-*cis*) isomers **1′** (Fig. 1) along with a total of 16 conformational isomers with favorable energies. t*Z*c conformation has been stated to be the thermodynamically more stable structure, however, the c*Z*c form is more suited structurally to lead to the formation of **1**. Hence, the isomerization of **1** should lead first to the generation of the c*Z*c form of **1**′, and then an equilibrium is setup for all the 16 possible conformers. Reverse thermal antarafacial [1,7] H-shift is responsible for the generation of **1′**^7–9^. Further confirmation of the pre-vitamin D2 structure is provided by the corresponding upfield shift of H6′ (*δ* = 5.95 ppm, ^3^*J* = 11.89 Hz, H7′ (*δ* = 5.69 ppm, ^3^*J* = 12.03 Hz) and the downfield shift observed for H9′ (*δ* = 5.51 ppm, m). Furthermore H3′ (m, *δ* = 3.92 ppm), almost merging with H3 of **1**, and the CH_3_ singlet at δ = 0.73 ppm, absolutely confirm the identity of **1′** (Fig. 1). We also observed a multiplet at *δ* = 1.81‒1.88 ppm, which can be assigned to the 2*α′* and 6*β′* protons (confirmed by 2D-NOESY). Additionally, no evidence of the corresponding 3-epimer of **1** was observed in the 2D-TOCSY and JRes spectra.

**Figure 2:**
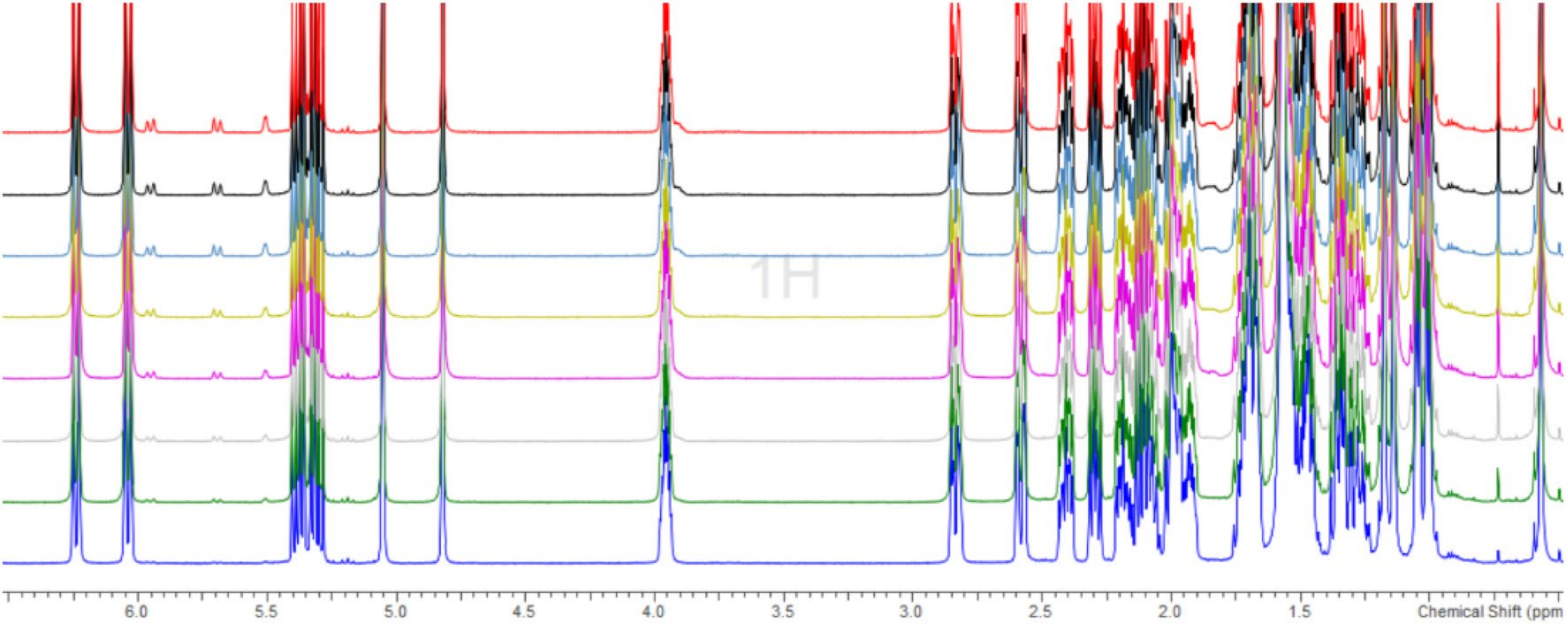
Overlay of 8 measurements at intervals of 8 h, for 25-hydroxyvitamin D2 (1).

The rate of disappearance of **1** is at a maximum of 0.89% and a minimum of 0.63%, absolute content wise, in the span of 56 h. An average estimation of this rate would be approx. 0.79% every 8 h. Since the isomerization of **1** to **1′** is in a state of thermodynamic equilibrium, the quantification of the corresponding appearance of **1′** was not performed, owing to a low signal-to-noise ratio. As can be seen in Fig. 2, at least a 0.80% decrease in the ‘count’ of **1** should be expected within 8 h at RT, when it is in solution.

### 24(*R*),25-Dihydroxyvitamin D2

Dihydroxyvitamin **2**, the R-isomer, is an important quantitation parameter for assessing the genetic inactivations of CYP24A1, therefore, we also studied the stability of solutions, after quantification with a similar qNMR method as mentioned for **1**. For quantification ca. 2.16 mg of **2** was weighed in a HPLC-vial together with 1,2,4,5-tetrachloronitrobenzene (qNMR internal standard; ca. 1.06 mg). To this mixture was added CDCl_3_ (ca. 190 µL, pH neutral), and the solution was transferred to a 3 mm NMR tube. A single qNMR experiment was performed using the methodology utilized for **1**, owing to the current limited availability of **2**. H6 was also utilized here for quantification, since it is the only signal free from interferences. The absolute content of **2** was found to be 93.46%. The rate of disappearance of **2** is at a maximum of 1.68% and a minimum of 0.3%, absolute content wise, in a span of 48 h. An average estimation of this rate would be approx. 0.89% every 8 h. This results in a total loss of 5.37% in 48 h.

This solution was, then, utilized for studying the stability within a span of 48 h. Quite similar to what was observed for **1**, molecule **2** also underwent isomerization, mainly, to pre-vitamin **2′**^19,20^. Similarly to **1**, corresponding upfield shift of H6 (*δ* = 5.95 ppm, ^3^*J* = 11.74 Hz) and H7 (*δ* = 5.69 ppm, ^3^*J* = 12.03 Hz) was observed. Additionally, the new methyl group, 2*α′* and 6*β′* protons are found at similar delta values, like pre-25-Hydroxyvitamin D2 **1′**. However, an upfield shift was observed for H9′ at *δ* = 5.51 ppm, when compared to H9 of **2**.

**Figure 3:**
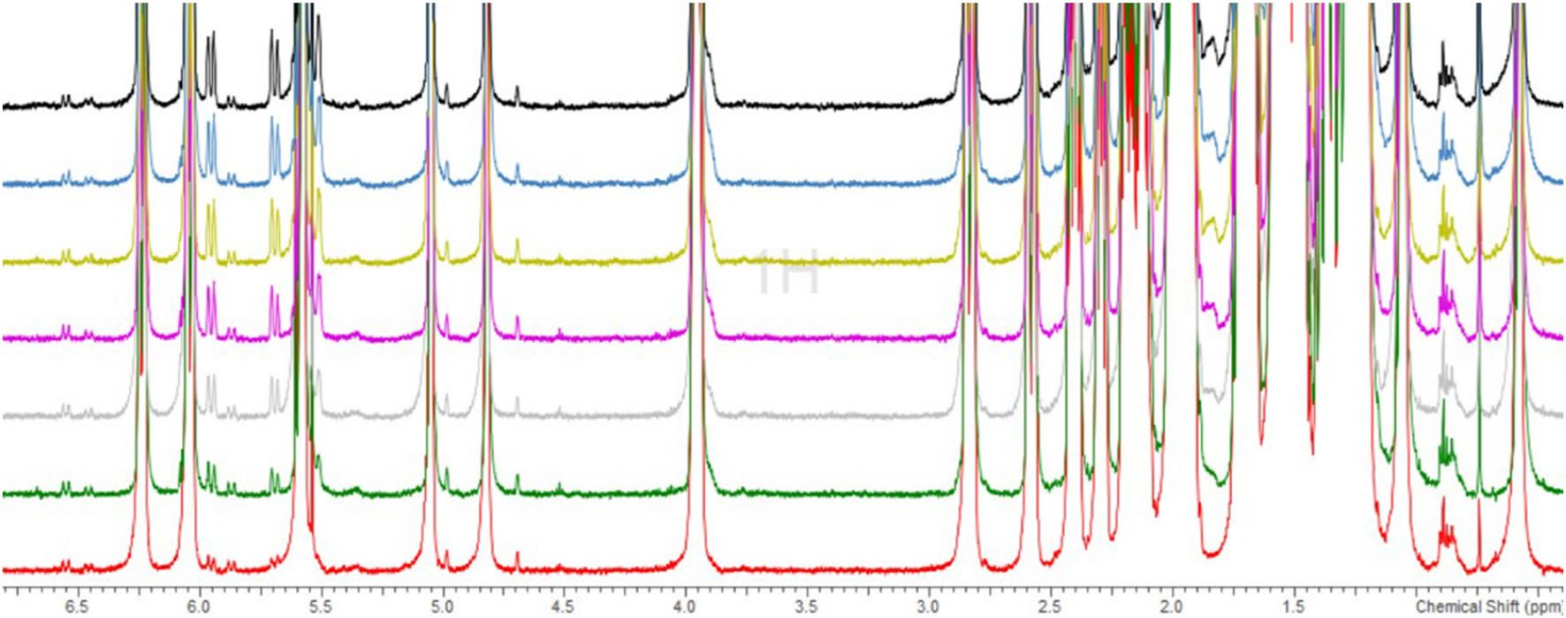
Overlay of 7 measurements at interval of 8 h, for 24(*R*),25-dihydroxyvitamin D2 (2), highlighting the nature of impurities.

Furthermore, as can be seen in Fig. 3, besides characteristic pre-vitamin **2′** signals, doublets are present at *δ* = 6.54 ppm, *δ* = 5.87 ppm, along with broad singlets at *δ* = 4.98 ppm and *δ* = 4.69 ppm. These signals can be assigned to the 5,6-*trans* isomer^21–23^ (5*E*,7*E*,22*E*-) **3** of **2**. Compounds like **3** can result from the synthetic route when vinyl-allenes^24^ are utilized as precursors for vitamin D analogues. Additionally, doublets are present at *δ* = 6.46 ppm, *δ* = 6.04 ppm (overlap) along with a signal shoulder can be seen at *δ* = 5.62 ppm overlapping with H9′ of **2**. These signals can be, probably, assigned to the corresponding lumisterol-type isomers^25^. However, these signals remain constant, when compared to the internal standard, and do not interfere with our quantitation method.

Molecule **2** shows the distinct analytical capabilities of qNMR, wherein careful selection of quantitation signals, based on the impurity-profile and stability studies, can actually deliver unparalleled results when compared to other analytical techniques, in a single non-destructive experiment.

### 25-Hydroxyvitamin D3

The absolute content determination for **4** was carried out, in a similar way, to compounds **1** and **2**. H6 was utilized as the quantification signal. For absolute content determination, 1.81‒2.49 mg of **4** was weighed together with the qNMR internal standard (1,2,4,5-tetrachloronitrobenzene; 1.04‒1.36 mg) on an ultra-micro weighing balance. To this mixture was added CDCl_3_ (ca. 190 µL, pH neutral), and the solution was transferred to a 3 mm NMR tube. Triplicate measurements were performed to determine the avg. absolute content of **4**, which yields a value of 91.84% (Table 1).

**Figure 4:**
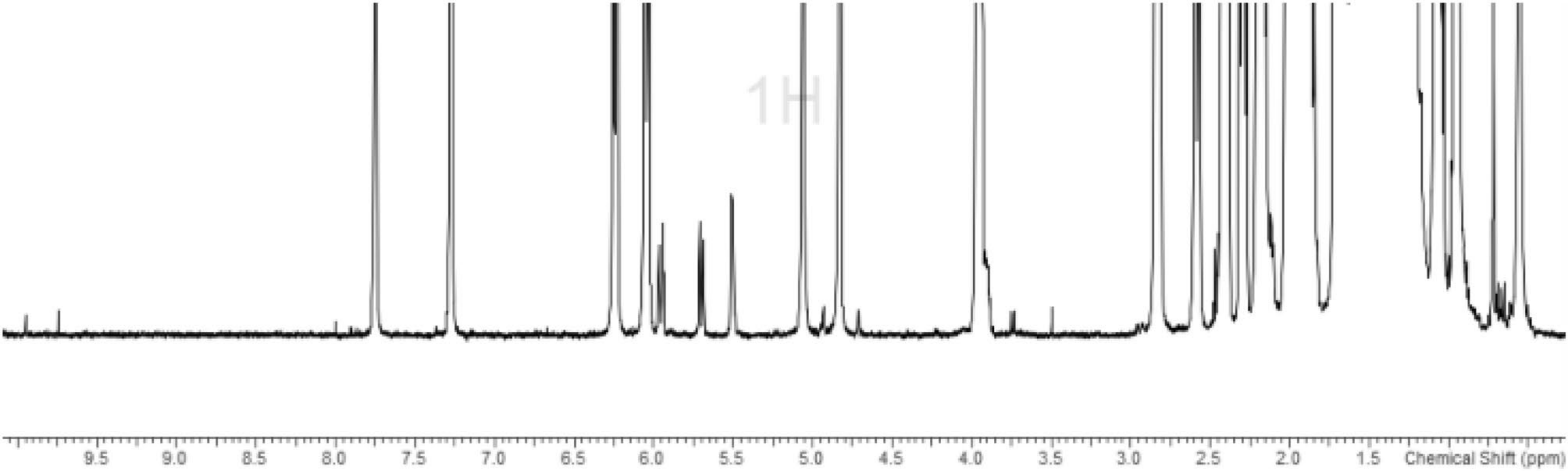
New signals after 56 h in the CDCl_3_ solution of molecule 25-hydroxyvitamin D3 (4).

Similar methodology was employed for the stability studies for 25-hydroxyvitamin D3 (4) within a time span of 56 h. Main isomerization product was identified, on the basis of diagnostic signals and literature values, to be the pre-vitamin **4′** (Fig. 4)^19–22^. A total of 5.92% reduction was observed in the absolute content of **4** within 56 h. However, additional growing signals, in trace amounts, at *δ* = 9.96 ppm (s), *δ* = 9.74 ppm (s), *δ* = 3.74 ppm (d; ^3^*J* = 8.31 Hz), *δ* = 2.24 ppm (dd; ^3^*J* = low resolution), *δ* = 2.11 ppm (m) and 3 singlets in ca. 0.65‒0.69 ppm range, can be observed. The precise structure of above mentioned signals is hard to asses owing to the extremely low quantity of these molecules, but this experiment sheds light on the fact that along with the main isomerization product (pre-vitamin), with time, many more impurities might emerge, maybe as a result of trace impurities present in the solvents used. Deuterated NMR solvents are generally of very high-grade but trace amount of metal catalysts along with dissolved oxygen/water cannot be completely ruled out. This can lead to further hydroxylations and formation of aldehydes. The stability of molecule **5** has been analyzed elsewhere, but in smaller amounts (µg/g) by LC-UV method in polar solvents, leading to the characterization of only pre-vitamin 4^26^.

Furthermore, no evidence of the formation/presence of pro-vitamin D **5a** and/or suprasterols I/II^27^ was found in any of the above examined vitamin D analogues.

### Choice of quantitation signal

**Figure 5:**
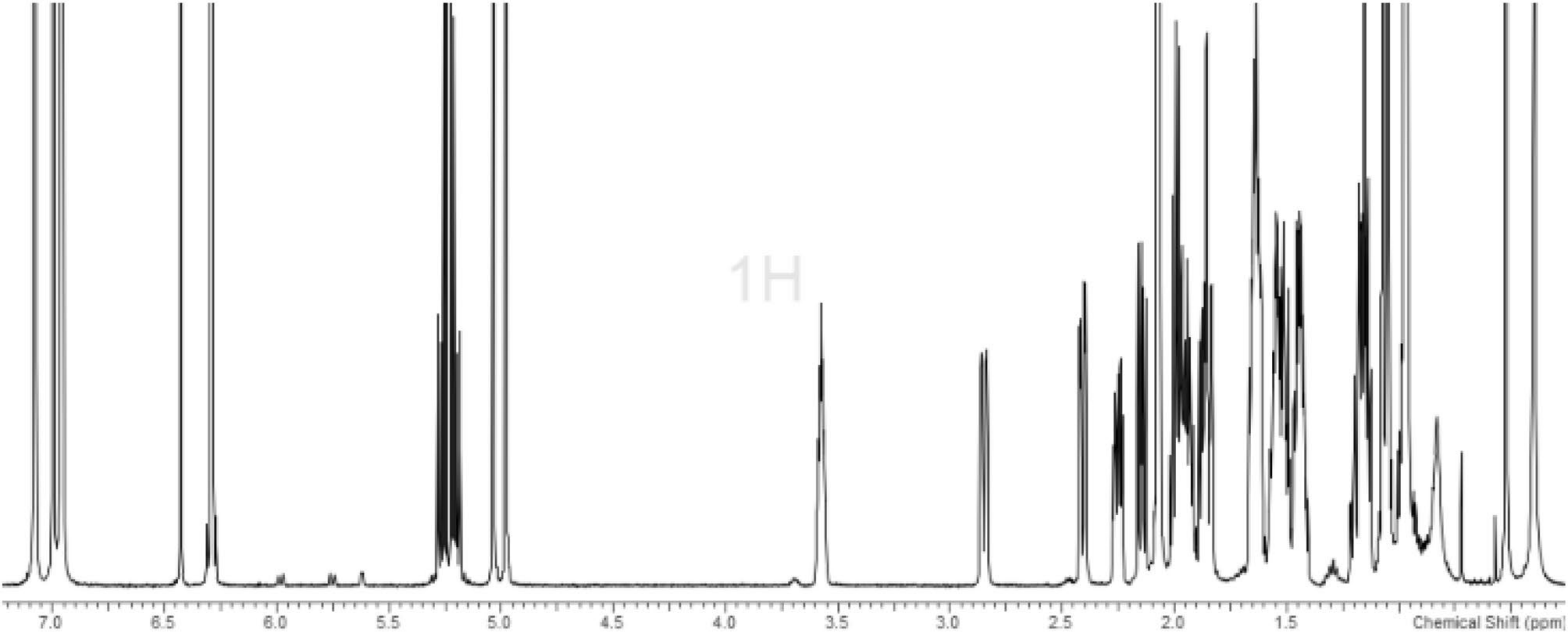
^1^H NMR spectrum of **1** in toluene-d_8_.

The advantage of utilization of H6 as the quantitation signal is that most of the impurities and/or the isomers present, as seen in above mentioned experiments, do not overlap with H6. However, it must be added that the presence of 3-epimer might lead to slightly overestimated results. Therefore, we also performed a qualitative ^1^H NMR of molecule **1** in toluene-d_8_ (Fig. 5). Aromatic solvent induced shifts (ASIS) can lead sometimes to better dispersion of signals owing to the interaction of the diamagnetic current of the benzene ring. A considerable effect was observed on the splitting pattern of H6 and H7. The doublet or dt splitting collapsed to an AB*q* pattern with *δ* = 6.30 ppm. This reflects a *∆δ* = 0.05 ppm for H6 and *∆δ* = 0.26 ppm for H7, when compared to the value in chloroform. Also, significant effect was observed on the 3H proton which witnessed a *∆δ* = 0.37 ppm. Therefore a change of stereochemistry here, for the 3-epimer, should manifest itself by a notable change in the chemical shift of 3H proton.

## Summary

It has been shown that the rate of disappearance of 25-hydroxyvitamin D2 (1), 24(*R*),25-dihydroxyvitamin D2 (2) and 25-hydroxyvitamin D3 (4) is 5.46%, 5.37% and 5.92%, respectively. Although these values should not be same in every solvent, they do correspond to the fact that at least 6% loss should be expected if solutions of these molecules are left at RT for a period of 56 h. This can make a significant difference if the loss %age is not accounted for making callibrators for LC, LC–MS/MS and/or GC–MS methods. Analytical labs where cooled-thermostatted auto-samplers are not available, it is recommended to include the loss %age in the final quantification. We have also shown that apart from pre-vitamin D formation, other molecules are also generated, with similar basic molecular structure, either through further transformations of pre-vitamin D or by interaction with possible trace impurities present in the solvents. Therefore, for highly precise quantifications of vitamin D analogues, high grade solvents/eluents (e.g., free from trace metal impurities. dissolved oxygen, water, etc.) should be utilized. Furthermore, H6 from the hexatriene system is suitable for quantification for all the three analytes when the presence of the corresponding 3-epimers has been ascertained in ASIS solvents.

## Supplementary Information

Supplementary Information.
