# Supplementary material for: Quantitative NMR (qNMR) spectroscopy based investigation of the absolute content, stability and isomerization of 25-hydroxyvitamin D2/D3 and 24(R),25-dihydroxyvitamin D2 in solution phase
Source: Sci Rep. 2022 Feb 22;12:3014. doi: 10.1038/s41598-022-06948-4 (PMC8863798; doi:10.1038/s41598-022-06948-4)
Supplement: Supplementary file 1 — Supplementary Information. [file 41598_2022_6948_MOESM1_ESM.docx]

**Quantitative NMR (qNMR) spectroscopy based investigation of the absolute content, stability and isomerization of 25-Hydroxyvitamin D2/D3 and 24(*R*),25-dihydroxyvitamin D2 in solution phase**

*Neeraj Singh,* Judith Taibon, Stephan Pongratz and Christian Geletneky**

*Roche Diagnostics, Nonnenwald 2, 82377 Penzberg, Germany*

*Email: neeraj.singh.ns1@roche.com, christian.geletneky@roche.com*

Table of Index

[1] Calculation of Absolute Content of 25-Hydroxyvitamin D2, 24(R),25-Dihydroxyvitamin D2 and 25-Hydroxyvitamin D3: 1

[2] Stability data for 25-Hydroxyvitamin D2, 24(R),25-Dihydroxyvitamin D2 and 25-Hydroxyvitamin D3: 2

[3] 1D and 2D-NMR spectra of 25-Hydroxyvitamin D2 for analyses purpose: 3

[4] 1D and 2D-NMR spectra of 24(*R*),25-Dihydroxyvitamin D2 for analyses purpose: 5

[5] 1D and 2D-NMR spectra of 25-Hydroxyvitamin D3 for analyses purpose: 7

## [1] Calculation of Absolute Content of 25-Hydroxyvitamin D2, 24(R),25-Dihydroxyvitamin D2 and 25-Hydroxyvitamin D3:

The following formula was utilized to calculate the absolute content of the vitamin D analytes:

$$Px\boldsymbol{=}\frac{Ix*Nstd*Mx*mstd}{Istd*Nx*Mstdmx}*Pstd$$

*Px* : Purity of the analyte (Analyte) as mass fraction; *Pstd* : Purity of the internal qNMR standard as mass fraction; *Ix* : Integral of the analyte; *Istd* : Integral of the internal standard; *Nx* : No. of analyte protons; *Nstd* : No. of internal qNMR standard protons; *Mx* : Molecular weight of the analyte; *Mstd* : Molecular weight of the qNMR internal standard; *mx* : Mass of analyte; *mstd* : Mass of qNMR internal standard

The following values are utilized for the qNMR measurements:

Molecular Weight of 25-Hydroxyvitamin D2 = 412.6477 g/mol; Molecular Weight of 25-Hydroxyvitamin D3 = 400.6371 g/mol; Molecular Weight of 24(R),25-Dihydroxyvitamin D2 = 428.6472 g/mol; Molecular weight of the qNMR internal standard ISTD (Tecnazene = 260.890 g/mol; Purity of the qNMR internal standard as mass fraction = 99.82%; Number of Protons taken into account, for both the ISTD and Analyte = 1 each.

## [2] Stability data for 25-Hydroxyvitamin D2, 24(R),25-Dihydroxyvitamin D2 and 25-Hydroxyvitamin D3:

## [3] 1D and 2D-NMR spectra of 25-Hydroxyvitamin D2 for analyses purpose:


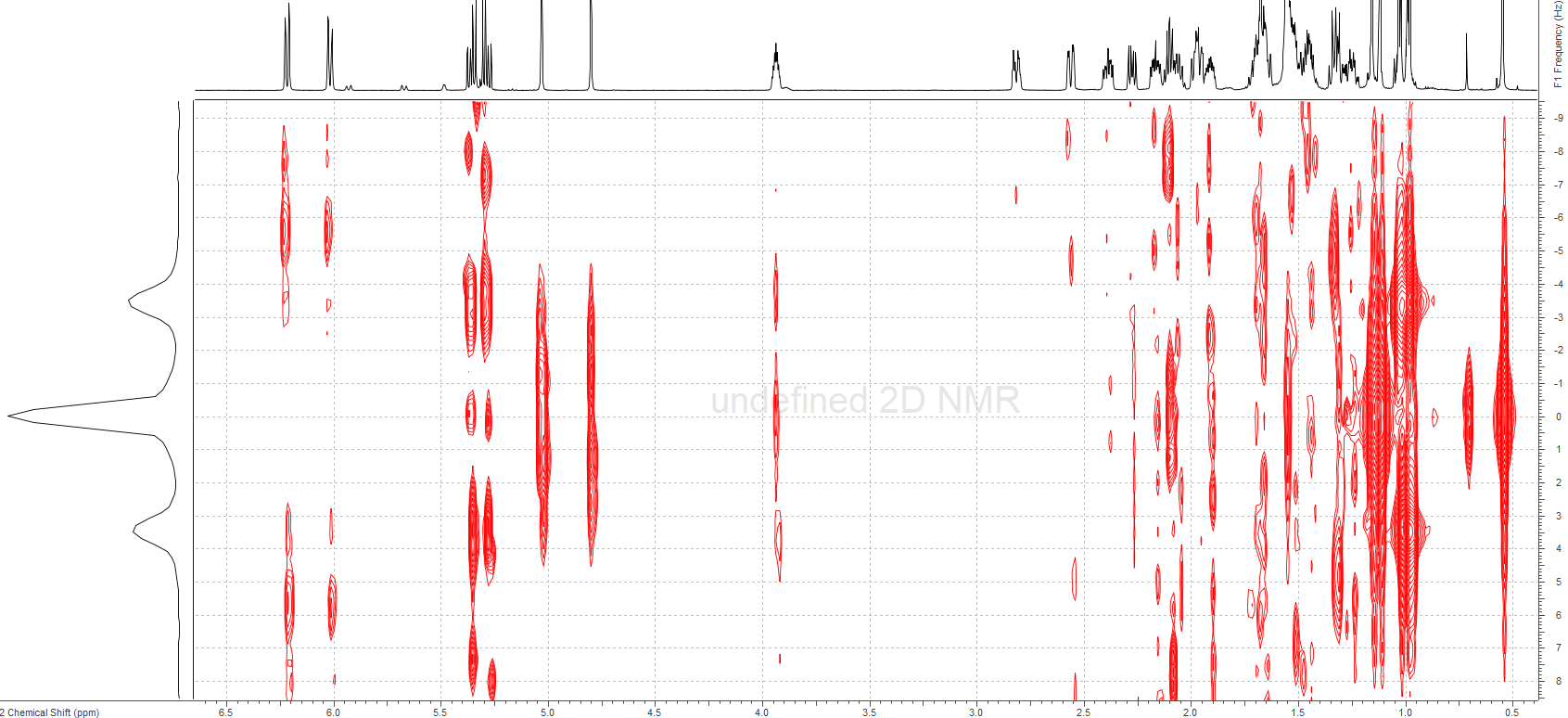


**Supplementary Figure 1**. *J*_Res_ spectrum for the elucidation of the coupling constants in 25-hydroxyvitamin D2


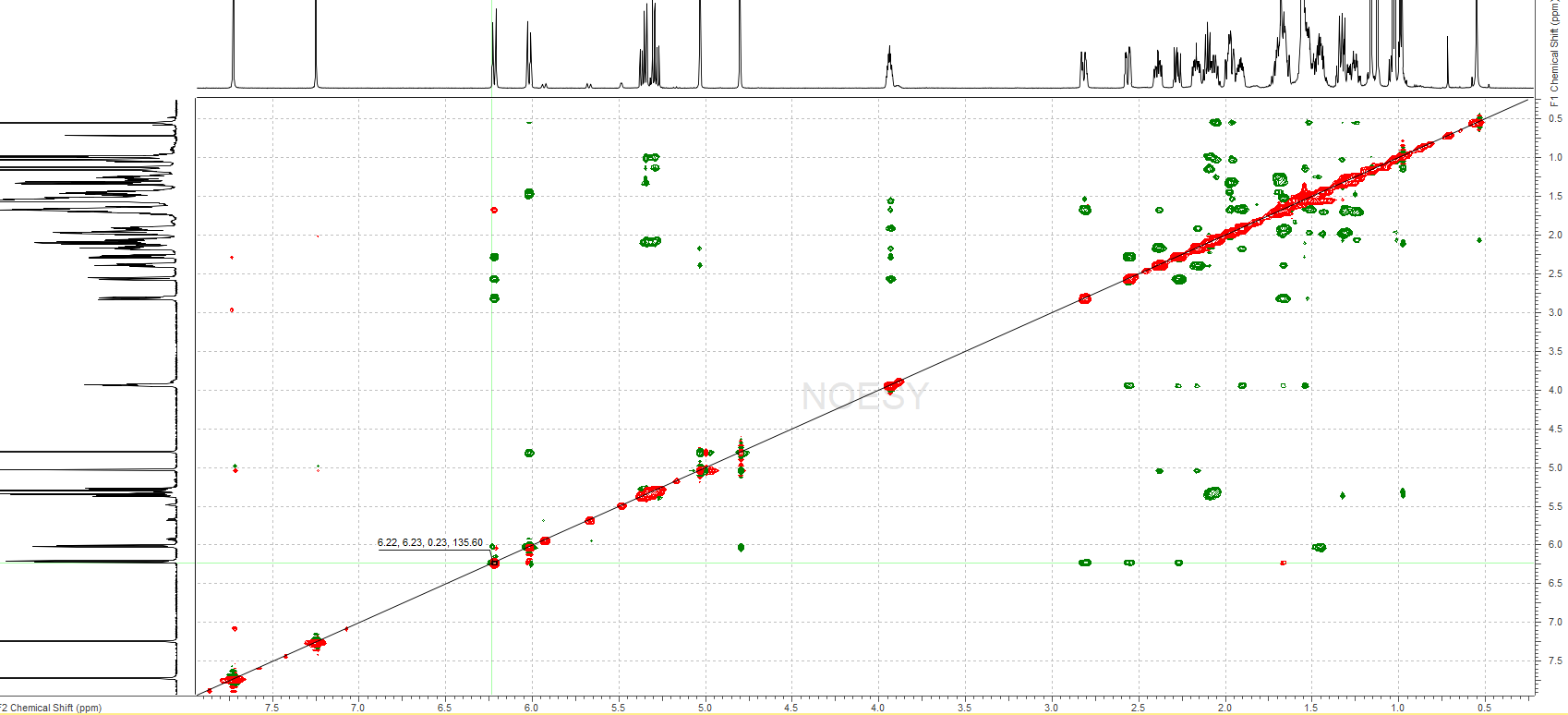


**Supplementary Figure 2**. 2D-NOESY_PHASE spectrum of 25-hydroxyvitamin D2


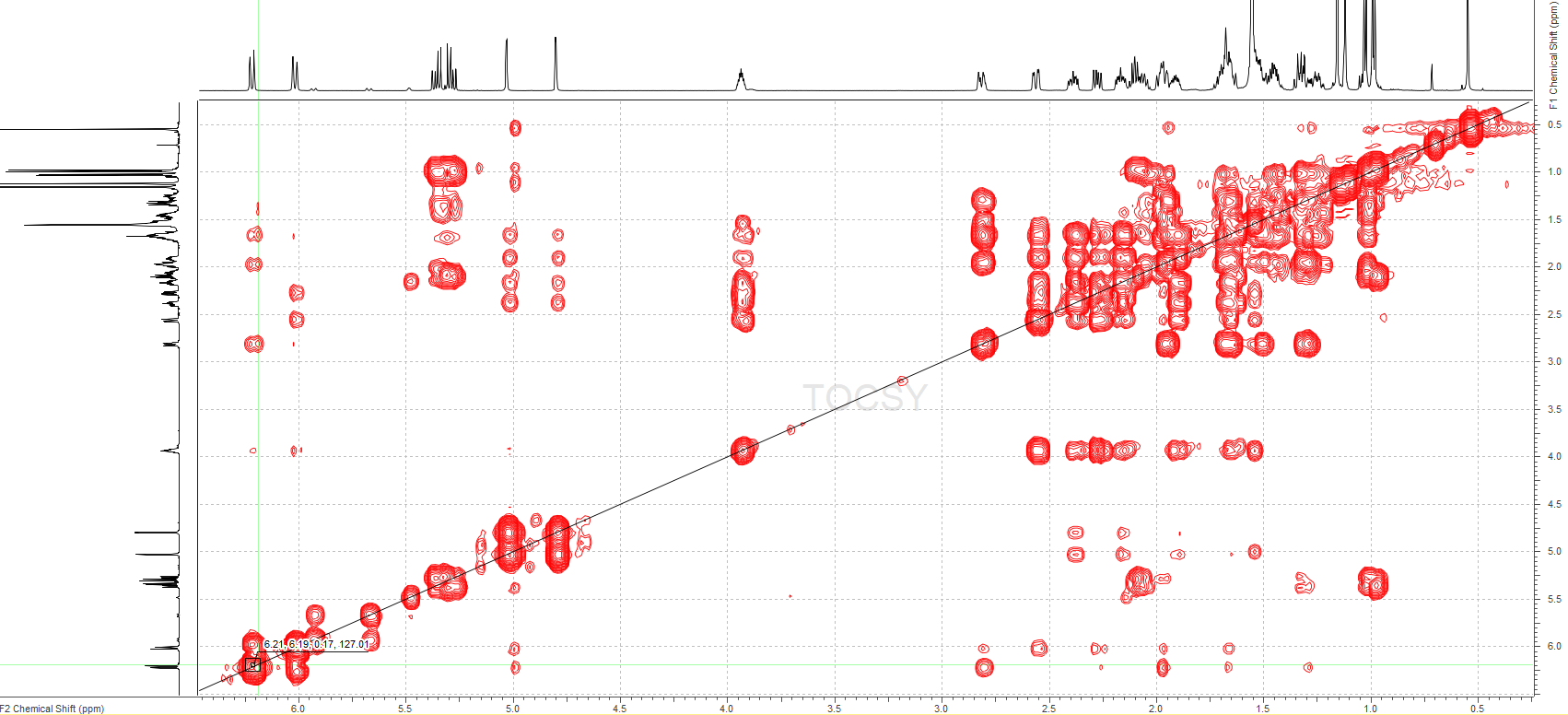


**Supplementary Figure 3**. 2D-TOCSY of 25-hydroxyvitamin D2

## [4] 1D and 2D-NMR spectra of 24(*R*),25-Dihydroxyvitamin D2 for analyses purpose:


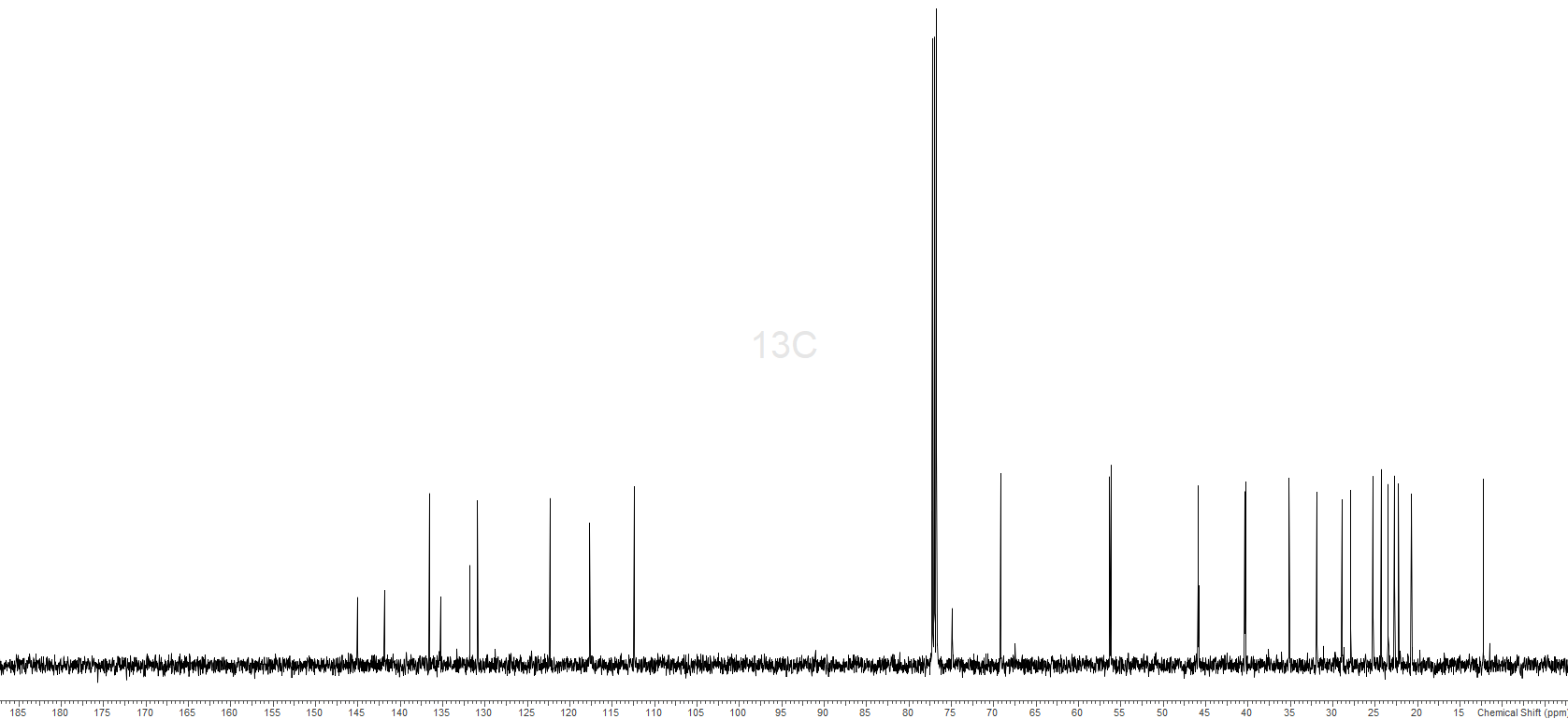


**Supplementary Figure 4**. ^13^C{^1^H}NMR of 24(*R*),25-dihydroxyvitamin D2


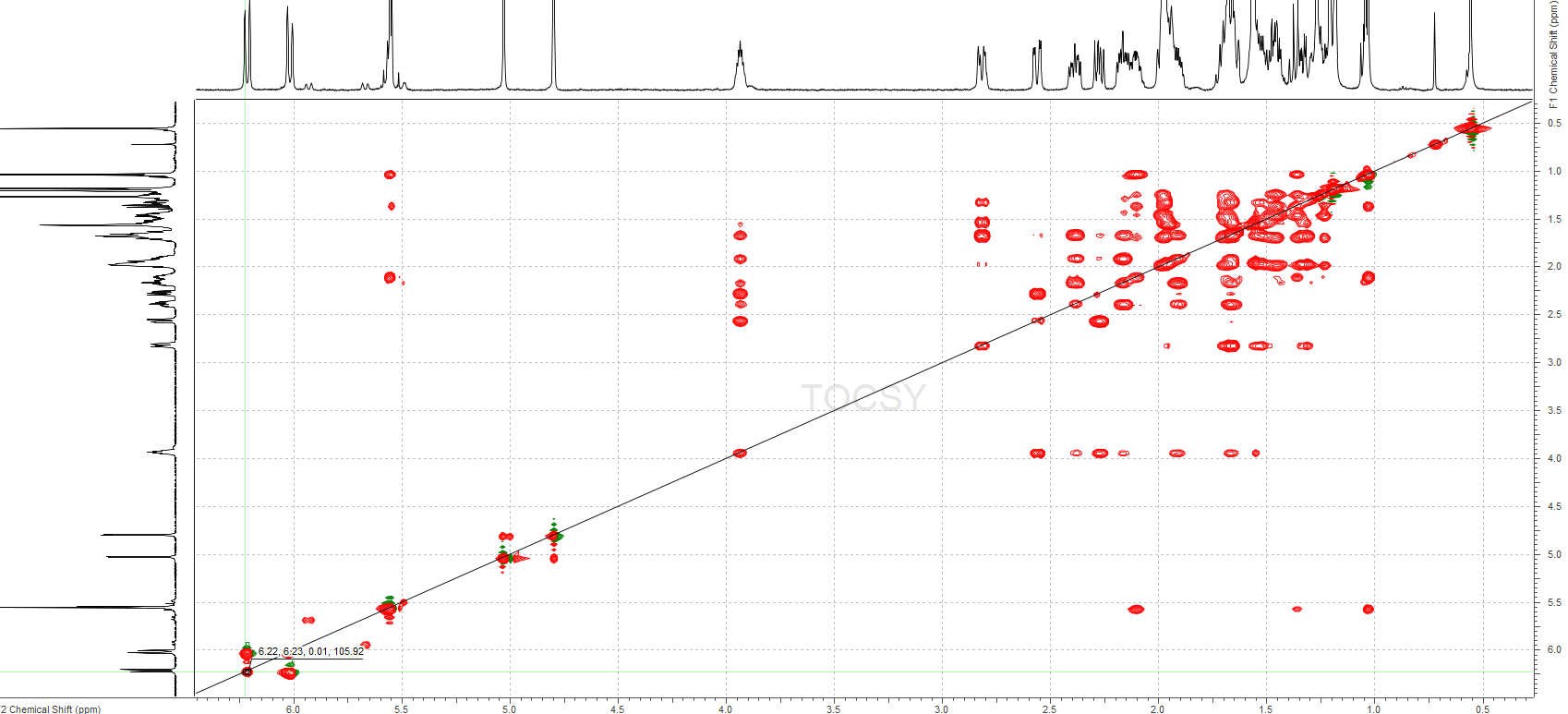


**Supplementary Figure 5**. 2D-TOCSY of 24(*R*),25-dihydroxyvitamin D2


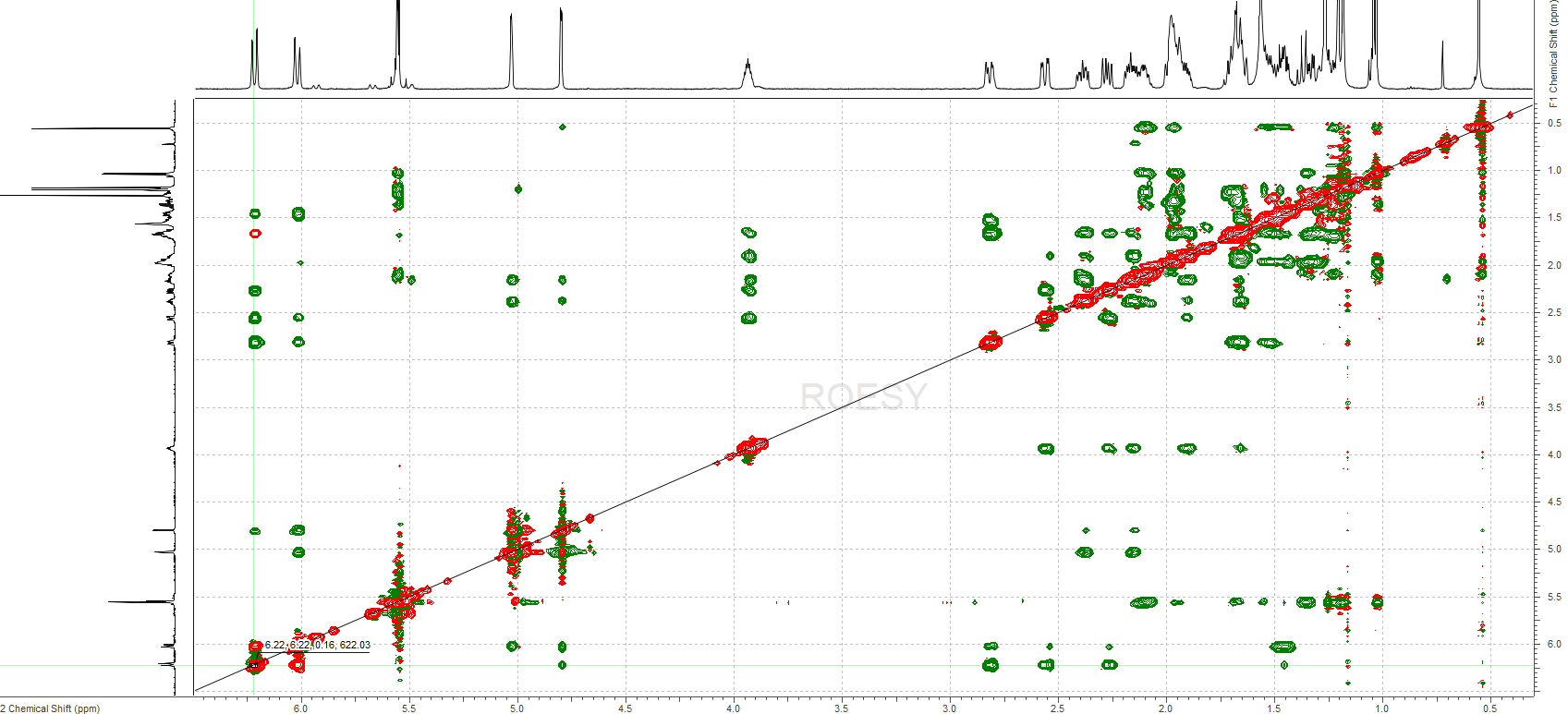


**Supplementary Figure 6**. 2D-ROESY_PHASE of 24(*R*),25-dihydroxyvitamin D2


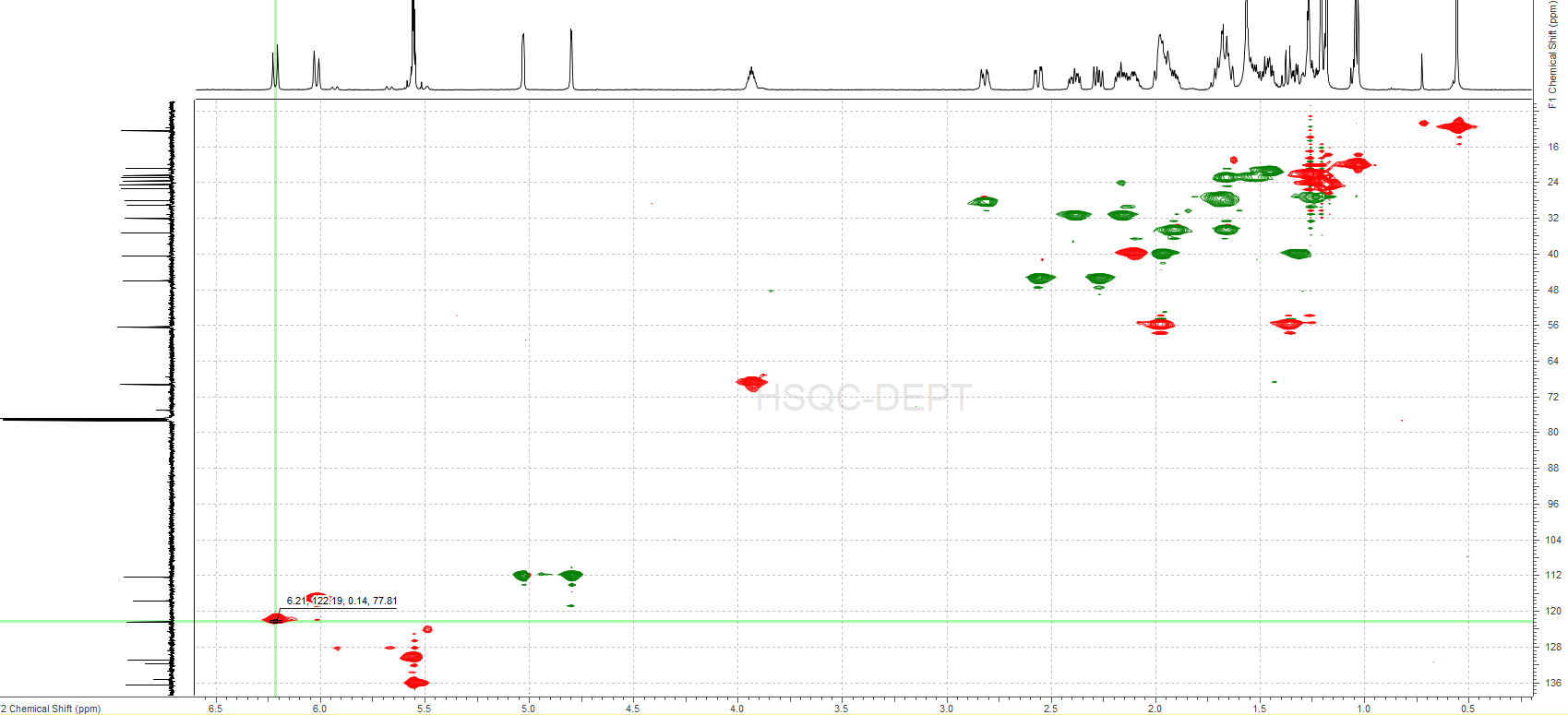


**Supplementary Figure 7**. gHSQCAD of 24(*R*),25-dihydroxyvitamin D2

## [5] 1D and 2D-NMR spectra of 25-Hydroxyvitamin D3 for analyses purpose:


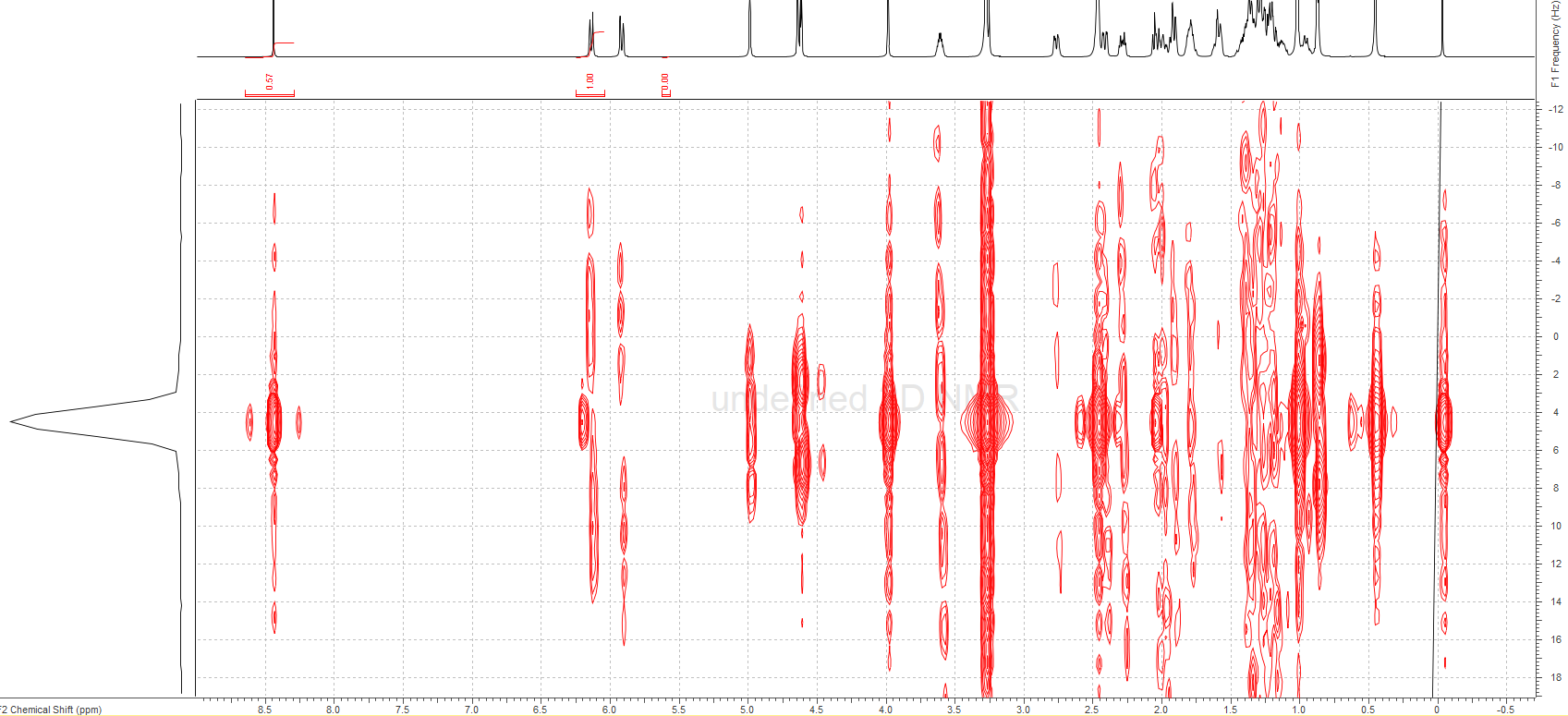


**Supplementary Figure 8**. *J*_Res_ spectrum for the elucidation of the coupling constants in 25-hydroxyvitamin D3


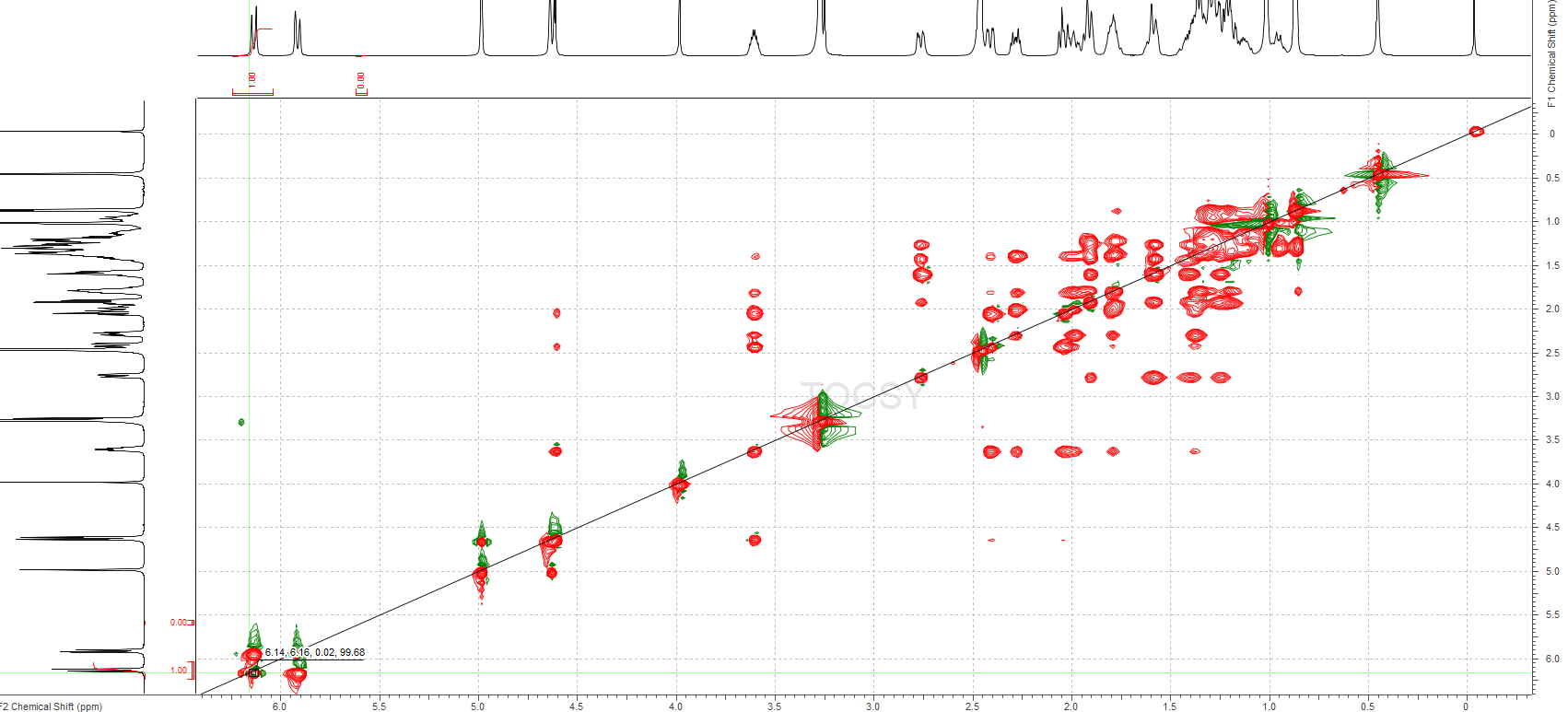


**Supplementary Figure 9**. 2D-TOCSY of 25-hydroxyvitamin D3
